# Anatomical Risk Factors for Lower Extremity Musculoskeletal Disorders and Proposed Prevention Strategies

**DOI:** 10.1298/ptr.R0040

**Published:** 2026-02-10

**Authors:** Junya OZAWA

**Affiliations:** Department of Rehabilitation, Faculty of Rehabilitation, Hiroshima International University, Japan

**Keywords:** Bone geometry, Malalignment, Musculoskeletal disorder, Joint unloading

## Abstract

Knee osteoarthritis (OA), patellofemoral pain, and anterior cruciate ligament (ACL) injuries are major musculoskeletal disorders often treated through conservative or postoperative physical therapy. Patellofemoral pain syndrome and patellar instability have been linked to femoral morphology specifically femoral anteversion (proximal) and trochlear groove (distal), as well as patellofemoral joint malalignment. Knee OA has been associated with an increased medial proximal tibial angle, indicating tibial varus alignment. ACL injuries are linked to abnormal bony morphology, including femoral intercondylar width, alpha angle, and posterior tibial slope angle. Preventing these abnormalities may reduce the risk of future diseases. Recent animal studies have shown that insufficient mechanical loading during growth alters bone geometry and joint alignment, which are risk factors for these musculoskeletal disorders. This review summarizes the bone morphological abnormalities and joint malalignments implicated in major lower limb musculoskeletal disorders, which have not been traditionally considered as therapeutic targets.

## Introduction

**Fig. 1.**
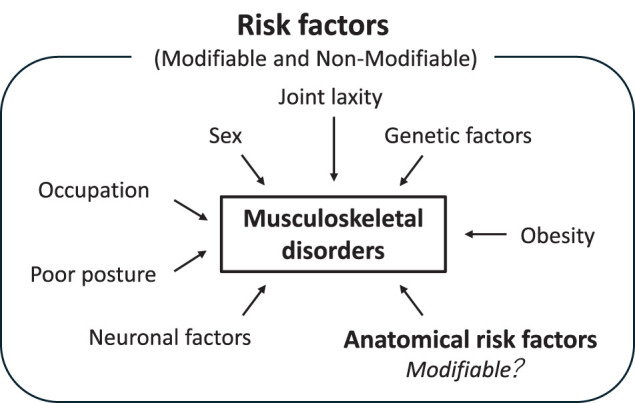
A conceptual model of musculoskeletal disorders in the lower extremities. Both modifiable and non-modifiable systemic and mechanical risk factors interact to promote the development of musculoskeletal disorders, including knee osteoarthritis, patellofemoral pain/instability, and anterior cruciate ligament injuries.

Patellofemoral pain syndrome, hip and knee osteoarthritis (OA), and anterior cruciate ligament (ACL) injuries are major musculoskeletal disorders frequently treated with either conservative or postoperative physical therapy. However, effective prevention strategies for these conditions have yet to be established, highlighting the need for preventive strategies. These musculoskeletal diseases are multifactorial^1–3)^, involving intrinsic, extrinsic, modifiable, and non-modifiable factors (Fig. 1). Among these factors, anatomical abnormalities have been considered key risk factors because of their ability to alter joint kinematics and the mechanical stress within the joint, potentially promoting the development and/or progression of musculoskeletal disorders.

Bone formation progresses rapidly from the fetal period until epiphyseal plate closure, after which it remains relatively unchanged. Therefore, bone characteristics formed during growth are maintained into adolescence and middle age, when musculoskeletal diseases are more prevalent. Several recent animal studies have demonstrated that insufficient mechanical loading on joints during growth can alter bone geometry and joint alignment. Such morphological abnormalities have been associated with risk factors for various musculoskeletal disorders, suggesting that appropriate mechanical loading on the lower extremities during childhood may modify anatomical risk factors.

This review summarizes bone morphological abnormalities and malalignments implicated in several major lower limb musculoskeletal disorders, including knee OA, patellofemoral pain/patellar instability, and ACL injuries. Furthermore, this paper introduces animal models demonstrating that altered loading during growth can induce bone morphological abnormalities and malalignments.

## Relationship Between Bone Morphology and Joint Disease

### Knee OA

Knee OA is a progressive disorder affecting the entire joint, including the articular cartilage, subchondral bone, synovium, meniscus, and ligaments. It has been associated with lower limb deformities and malalignments, particularly tibiofemoral malalignment in the frontal plane. One systematic review revealed that patients with knee OA in Asia and Europe predominantly exhibit varus deformity (Type I in the Coronal Plane Alignment of the Knee classification)^4)^. Another systematic review identified knee malalignment as an independent risk factor for knee OA progression^5)^. The progression of knee OA is accompanied by an increased joint line convergence angle, an index of tibiofemoral joint congruity, decreased hip–knee–ankle angle, an index of varus/valgus knee, and decreased medial proximal tibial angle (MPTA), the angle between the axis of the tibia and the proximal tibial joint surface^6)^, in the coronal plane. In contrast, other studies have reported that abnormal femoral, but not tibial, geometry is associated with varus OA^7)^. Furthermore, research has shown that the mechanical lateral distal femoral angle (mLDFA), which increases in valgus OA^8)^, decreases in varus OA, whereas MPTA remains unchanged^9)^. Thus, whether varus deformity arises from the proximal tibia, distal femur, or both remains unclear.

### Patellofemoral pain/patellar instability

Patellofemoral pain is among the most common causes of knee pain in young adults^10)^. Several risk factors have been associated with patellofemoral pain, including patellar maltracking, imbalance between the vastus medialis and lateralis, and hip instability^1)^. These factors increase the stress placed on the patellofemoral joint, thereby promoting pain and symptom aggravation. Abnormal patellofemoral joint alignment can also increase joint stress.

One meta-analysis revealed that several patellofemoral malalignments, including a larger bisect offset and congruence angle (indicating lateral patellar displacement), larger patellar tilt angle (indicating greater lateral tilt), and lesser patellofemoral contact area^11)^ are associated with the presence of patellofemoral pain. Furthermore, another study showed that specific patellofemoral malalignments, including a higher Insall–Salvati ratio (indicating patella alta), larger patellar tilt angle, and larger bisect offset in people with patellofemoral pain, were associated with the presence of abnormal cartilage, bone, and soft tissue on magnetic resonance imaging^12)^.

Patellar instability is among the most common knee pathologies occurring during growth^13,14)^. Patellar dislocations can be divided into congenital, acute traumatic, and acute constitutional dislocations^13)^. Congenital patellar dislocations are rare, with 69% of the first patellar dislocations occurring between age 10 and 19^14)^. Numerous studies have identified abnormal bone morphology (trochlear dysplasia) and knee malalignment as major risk factors for lateral patellar instability. Indeed, evidence suggests that abnormal femoral morphology, such as increased femoral neck anteversion angle (AVA)^15,16)^, decreased trochlear depth^17)^, trochlear facet asymmetry^17)^, decreased lateral trochlear inclination (LTI)^18,19)^, a larger sulcus angle (angle between the medial and lateral facets)^17,19)^, and knee malalignment, (e.g., increased tibial tubercle–trochlear groove [TT–TG] distance^17,19)^) and patella alta^17)^, is associated with patellar instability. Moreover, computational simulation analysis showed that increased AVA^20)^ and TT–TG distance^21)^ are associated with a larger sulcus angle, which is an important factor contributing to patellar dislocation^22)^. These results indicate that multiple abnormal bone morphologies and knee alignment interact to promote the development of symptoms and diseases. Moreover, a systematic review concluded that trochlear morphology and knee alignment are associated with patellofemoral OA^23)^, indicating that patellofemoral pain and/or patellar instability may eventually progress to patellofemoral OA.

### ACL injury

ACL injuries are quite common, especially among young women who play sports such as soccer and basketball^3)^. The mechanisms by which ACL injuries occur can be classified into either contact or noncontact, with the latter being more common^24)^. Various risk factors for ACL injuries have been identified, including collagen-related single-nucleotide polymorphisms and biomechanical patterns, such as landing techniques and knee positioning^25)^. Several anatomical risk factors, such as femoral intercondylar notch width^26–28)^, dysplastic trochlear morphology^29–31)^, increased AVA^32)^, increased posterior tibial plateau slope^3,24,33,34)^, and increased α-angle (formed between the femoral axis and Blumensaat line)^35)^, have also been proposed. These bone morphological abnormalities are believed to increase susceptibility to ACL injuries by increasing tensile loading on the ACL and altering contact with the adjacent femoral condyle.

## Are Bone Morphological Abnormalities Present Prior to Disease Onset?

Higano et al.^36)^, who investigated the longitudinal changes in the tibial plateau angle in non-OA women, showed that the tibial plateau angle at baseline (average age 49.5 years) was significantly smaller in patients with advanced OA (Kellgren–Lawrence grade 3 or 4) than in patients without OA (grade 0 and 1) or early OA (grade 2) diagnosed 21 years later. This finding suggests that abnormal bone morphology may precede, rather than result from, disease development.

Bone morphological abnormalities and malalignments are traditionally considered intrinsic, non-modifiable risk factors for several musculoskeletal disorders^3)^. However, certain abnormalities may be preventable. For instance, developmental dysplasia of the hip (DDH) can occur with prolonged mispositioning of the hip joint (hip extension and adduction) during infancy^37)^. Therefore, lower incidence rates of DDH have been observed in populations practicing “hip-safe” techniques for infants than in those that do not^38)^, possibly due to the optimization of the intra-articular mechanical environment through proper joint positioning. Furthermore, studies have reported a decrease in the AVA until growth completion during childhood, indicating that the action of the hip muscles during gait may shape the AVA^39)^. These results suggest that mechanical stress on the joints during early childhood, when bone formation is incomplete, can alter bone morphology.

## Effects of Joint Loading on Bone Morphology in Animal Models

**Fig. 2.**
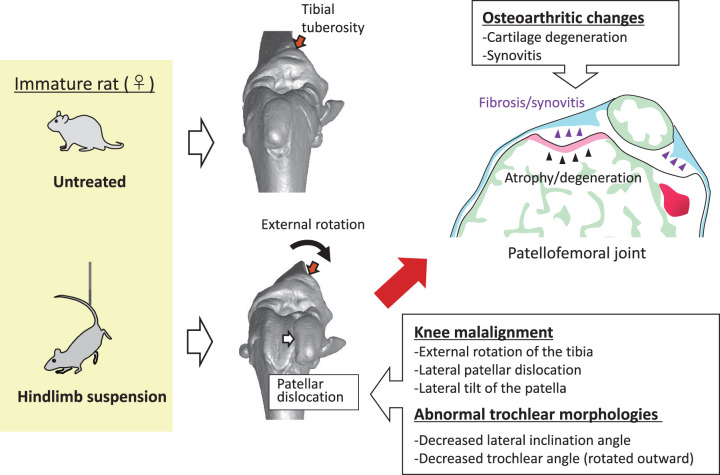
Effects of reduced weight-bearing during growth on the knee joint. Joint unloading can promote the development of abnormal trochlear morphology, patellofemoral and femorotibial malalignment, lateral patellar dislocation, and osteoarthritic changes.

Numerous studies have investigated the effects of unloading on bone mass^40)^, strength^41)^, mineral density^42)^, and microstructure in animal models^41,43)^. However, relatively few studies have focused on the effects of reduced loading, particularly during growth, on the geometric changes in the hindlimb bones. Ohira et al. reported that hindlimb suspension (HS) from postnatal day 4 to month 3 induces an irreversible external bend in the shaft and external rotation of the distal end of tibia^40)^. HS can affect bone morphologies associated with several musculoskeletal disorders even after acquiring locomotion. In 4-week-old rats, HS increased the AVA and promoted dysplastic changes in the femoral trochlear, such as a decreased LTI, decreased trochlear angle (outward rotation), and asymmetry in the medial/lateral condyle size based on 3-dimensional computed tomography^44)^. Moreover, studies have shown that HS during growth can induce knee joint malalignments, such as abnormal TT–TG distance and patellar tilt angle with patellofemoral OA and lateral patellar dislocation^45)^ (Fig. 2). If HS persists beyond a certain period (2 or 4 weeks), these bone morphological abnormalities and joint malalignment become irreversible^46)^. These abnormalities and associated abnormal locomotion patterns can persist even after prolonged reloading in rats (54-week-old)^47)^. These results suggest that insufficient weight-bearing during infancy may trigger the development of anatomical risk factors for musculoskeletal disorders, potentially promoting the onset of these diseases in the future.

Clinically, delayed or non-acquired motor development likely promotes decreased weight-bearing in early childhood. Patients with Down syndrome (DS) have a genetic anomaly and are characterized by physical, mental, and organismic alterations. Children with DS acquire motor skills through the same sequence (rolling, sitting, crawling, standing, and walking) as normal infants but do so at a later age^48,49)^. Patients with DS also present bone morphologies resembling those induced by unloading in animal models, including increased AVA^50)^, dysplastic knee^51)^, and lateral patellofemoral instability^52)^, which have been attributed to muscle hypotonia and ligamentous laxity. However, the aforementioned preclinical studies have shown that insufficient joint loading due to delayed motor development may also promote, at least in part, the development of bone morphological abnormalities and subsequent disorders in patients with DS.

## Future Perspectives

**Fig. 3.**
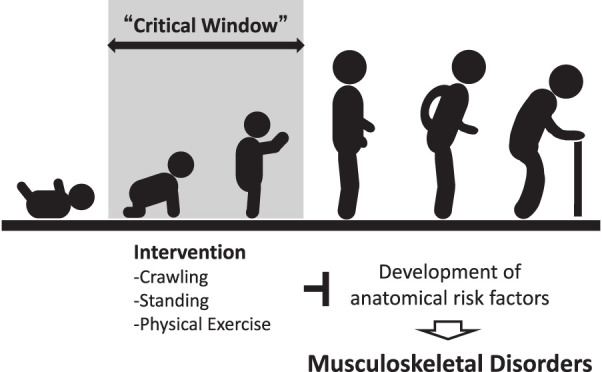
Schema illustrating strategies for preventing the emergence of anatomical risk factors for musculoskeletal disorders. Mechanical input to the joints during the “critical window” (i.e., infancy and early childhood) may be important for promoting joint structural integrity and resistance to musculoskeletal disorders.

Recent preclinical studies have shown that joint loading conditions during growth can alter bone morphology and alignment, previously regarded as non-modifiable, potentially promoting the development of disease. In other words, the growth period is a “critical window” for the formation of disease-resistant joint structures (Fig. 3). Therefore, appropriate weight-bearing exercises during growth, especially during early childhood, may prevent future musculoskeletal disorders. However, the optimal exercise conditions (e.g., timing, quantity, and quality) for preventing such disorders remain unclear, warranting further research before translation to human applications.
